# The Prevalence and Patterns of Menopausal Symptoms in Women Living with HIV

**DOI:** 10.1007/s10461-022-03696-4

**Published:** 2022-05-23

**Authors:** H Okhai, C Sabin, K Haag, L Sherr, R Dhairyawan, J Shephard, G Richard, F Burns, F Post, R Jones, Y Gilleece, S Tariq

**Affiliations:** 1 Institute for Global health, University College London, London, UK; 2 Department of Infection and Immunity, Barts Health NHS Trust, London, UK; 3 UK Community Advisory Board, London, UK; 4 Royal Free London NHS Foundation Trust, London, UK; 5 Kings College Hospital NHS Foundation Trust, London, UK; 6 Chelsea and Westminster Healthcare NHS Foundation Trust, London, UK; 7 Brighton & Sussex University Hospitals NHS Trust, Brighton, UK; 8 Brighton & Sussex Medical School, Brighton, UK

**Keywords:** HIV, Ageing, Menopause, Symptoms

## Abstract

Increasing numbers of women with HIV are experiencing menopause. We use data from a large, representative sample of women with HIV to describe the prevalence and clustering of menopausal symptoms amongst pre-, peri- and post-menopausal women using hierarchical agglomerative cluster analysis. Of the 709 women included, 21.6%, 44.9% and 33.6% were pre-, peri- and post-menopausal, respectively. Joint pain (66.4%) was the most commonly reported symptom, followed by hot flashes (63.0%), exhaustion (61.6%) and sleep problems (61.4%). All symptoms were reported more commonly by peri- and post-menopausal women compared to pre-menopausal women. Psychological symptoms and sleep problems clustered together at all menopausal stages. Somatic and urogenital symptom clusters emerged more distinctly at peri- and post-menopause. We recommend regular and proactive assessment of menopausal symptoms in midlife women with HIV, with an awareness of how particular patterns of symptoms may evolve over the menopausal transition.

## Introduction

In 2019, over 30,000 women living with HIV attended for HIV care in the UK, one in three (36.2%) of whom were aged over 50 [1]. Successes in lifelong antiretroviral treatment (ART) mean that women with HIV now have a similar life expectancy to their HIV-negative counterparts [2]. Therefore, this population is now increasingly experiencing age-related conditions including menopause.

Oestrogen depletion marks the beginning of the menopause transition and manifests in a range of symptoms, impacting physical and psychological well-being. It is estimated that 85% of women experience at least one symptom during the peri-menopause phase, which can persist post-menopausally [3]. The most prevalent symptoms reported are vasomotor (such as hot flashes and night sweats), however urogenital symptoms (including vaginal dryness, urinary symptoms and sexual dysfunction), mood changes, sleep disturbance, cognitive changes and joint pain can also be present [4].

As greater numbers of women with HIV reach midlife, evidence has accrued to suggest a high prevalence of menopausal symptoms in this population [5–9], with these symptoms potentially being more severe than in the general population [10, 11]. It is essential that women with HIV are aware of menopause and its associated symptoms [12]. This is particularly important in the context of a clinical condition associated with multi-morbidity. The potential overlap of numerous symptoms (including hot flashes/night sweats, exhaustion, sleep problems and depression) related to both HIV and/or menopause can make it hard for women and their healthcare providers to identify aetiology and implement appropriate management plans [13, 14].

To date, there has been limited information about women ageing with HIV, with existing published descriptive data primarily relating to age at menopause or prevalence of menopausal symptoms [15]. Understanding which symptoms cluster together, and their trajectory over the menopause transition, may provide insights into potential underlying causes and highlight how and when to screen and offer appropriate support to maintain the health and quality of life of women with HIV [16]. The Positive tRansItions through the MEnopause (PRIME) Study was designed to explore menopause amongst women living with HIV in England aged 45–60 years [17]. In this analysis, we use PRIME study data to describe the prevalence of menopausal symptoms and describe which symptoms commonly co-occur amongst pre-, peri- and post-menopausal women in a large, representative sample of women living with HIV.

## Methods

The PRIME Study was a cross-sectional, mixed-methods observational study of the impact of the menopause transition on the health and well-being of women with HIV. The study methods are described in detail elsewhere [17]. Briefly, women aged between 45 and 60 years were recruited from one of 21 National Health Service (NHS) HIV clinics across England between February 2016 and June 2017. Women were ineligible if they had experienced surgical menopause, had received chemotherapy or radiotherapy in the last six months, had used hormonal contraception within the last six months for either contraceptive or non-contraceptive use (women who had an intrauterine system inserted as part of hormone replacement therapy were included) or if their last menstrual period was more than 60 months prior to study enrolment. The PRIME Study had ethical approval from the South East Coast-Surrey Research Ethics Committee (REF 15/0735). All participants provided written informed consent.

Paper questionnaires were completed by all participants and included questions relating to: participant demographic/social factors (age, ethnicity, employment status, relationship status, highest level of education); current lifestyle factors (smoking, recreation drug use, alcohol use [assessed using the Alcohol Use Disorders Identification Test, AUDIT-C, with a score of ≥ 5 considered to be risky]); HIV-related (most recent clinical markers including CD4 T-cell count and HIV viral load) and non-HIV medical history (history of depression, diabetes, hypertension, cardiovascular disease, hepatitis B/C co-infection, breast cancer, osteoporosis, stroke) and menopause-related symptoms. The total number of medical conditions (not including HIV) reported by women (from the list above) was used as a proxy for multimorbidity.

Menopausal status was determined based on the modified Stages of Reproductive Aging Workshop (STRAW) + 10 criteria [18] (without biological confirmation) and was categorized as follows: pre-menopausal (menses within the past 3 months, no interval of amenorrhoea for ≥ 60 days in the past 6 months and no late period in the past 2 years); peri-menopausal (menses within the past 12 months but an interval of amenorrhoea for ≥ 60 days in the past 6 months and/or a late period in the past 2 years); and post-menopausal (amenorrhoea for ≥ 12 months).

Menopause-related symptoms were assessed using the validated Menopause Rating Scale, [MRS] which measures perceived severity of each of 11 symptoms in three domains: somatic [episodes of hot flashes/sweating, heart discomfort, sleeping disorders and joint/muscle complaints]; psychological [depression, irritability, anxiety and exhaustion]; and urogenital [sexual problems, vaginal dryness and urinary complaints]. For each symptom, perceived severity was scored on a Likert scale from 0 (symptom not present) to 4 (very severe). A composite score was calculated by summing the scores and categorized as: no/few (0–4), mild [5–8], moderate [9–16] and severe symptoms (≥ 17) [19].

Demographic, lifestyle and clinical characteristics of participants were described and compared by menopausal symptom severity using χ^2^ tests or Kruskal-Wallis tests, as appropriate. Thereafter, we described the prevalence of each of the 11 menopausal symptoms by menopausal status.

Clustering was based on responses to the severity of menopausal symptoms and was explored using hierarchical agglomerative cluster analysis of a dissimilarity matrix using wards-linkage (also known as minimal increase of sum-of-squares) [20]. Using this method we attempted to cluster symptoms instead of individuals, therefore creating a dendrogram which considers each symptom as a cluster of size one; it then joins similar clusters together using the Euclidean distance (the square root of the sum of the square differences) to visually identify which symptoms are commonly reported together. Symptom clusters were described separately among women of pre-, peri- and post-menopausal status, in order to explore whether the clustering of symptoms changed by menopausal stage. A shorter horizontal distance between clusters suggests a closer relationship between the symptoms. Statistical analyses were conducted in Stata (17.0, StataCorp LLC, College Station, TX).

## Results

A total of 868 women living with HIV participated in the PRIME study; of these, 709 (81.7%) completed the MRS questionnaire and therefore provided information on menopausal symptoms and were included in the analysis. Women who were included were more likely to be employed (68.9%) or in a relationship (57.1%) than those who were excluded (54.4% and 29.7%, respectively). In contrast, included women were less likely to report ever being diagnosed with depression than those who were excluded (30.7% vs. 42.9%).

The median age of the 709 women included was 49 [interquartile range (IQR): 47–52] years, the majority were of black African ethnicity (71.7%), currently employed (68.9%), had completed at least high school education (88.4%) and in a relationship (57.1%) (Table 1). Over one-third (36.6%) reported at least one medical condition (median: 0 [IQR: 0–1]; range: 0–3). Only 8.5% were current smokers, 14.2% reported hazardous drinking (AUDIT-C score ≥ 5) and 2.7% reported any recreational drug use in the last six months. In total, 21.6%, 44.9% and 33.6% of women reported being in the pre-, peri- and post-menopausal stages, respectively.

**Table 1:** Demographic, social and clinical characteristics relating to HIV and menopause reported by women from the PRIME study included in the analysis, stratified by severity of menopausal symptoms

Variables		All participants	Severity of menopausal symptoms (MRS scale)					p-value
			**No/little complaint** **(0–4)**	**Mild** **(5–8)**	**Moderate** **(9–16)**	**Severe** **(≥** **17)**	**Chi-squared (df)**	
N		709	211	117	182	199		
Age at completion of questionnaire, median (IQR)		49 (47, 52)	48 (46, 52)	49 (47, 52)	50 (47, 53)	50 (48, 53)	16.68	0.003
Menopausal status	Pre-menopausal	151 (21.6%)	80 (38.1%)	22 (19.0%)	33 (18.5%)	16 (8.2%)	56.32 (6)	< 0.001
	Peri-menopausal	314 (44.9%)	74 (35.2%)	53 (45.7%)	82 (46.1%)	105 (53.6%)		
	Post-menopausal	235 (33.6%)	56 (26.7%)	41 (35.3%)	63 (35.4%)	75 (38.3%)		
Ethnicity	Black African	500 (71.7%)	162 (78.6%)	89 (76.7%)	125 (70.2%)	124 (62.9%)	18.53 (9)	0.03
	White UK	90 (12.9%)	18 (8.7%)	10 (8.6%)	25 (14.0%)	37 (18.8%)		
	Black other	61 (8.8%)	18 (8.7%)	8 (6.9%)	14 (7.9%)	21 (10.7%)		
	Other	46 (6.6%)	8 (3.9%)	9 (7.8%)	14 (7.9%)	15 (7.6%)		
Currently employed		472 (68.9%)	160 (78.4%)	87 (78.4%)	135 (76.3%)	90 (46.6%)	62.46 (3)	< 0.001
In a relationship		386 (57.1%)	114 (57.3%)	59 (53.2%)	108 (61.7%)	105 (55.0%)	2.58 (3)	0.46
Completed education	Did not finish school	72 (10.6%)	20 (9.8%)	11 (9.8%)	16 (9.1%)	25 (13.4%)	11.98 (6)	0.06
	High school/equivalent	298 (43.9%)	84 (41.2%)	42 (37.5%)	76 (43.2%)	96 (51.3%)		
	University	309 (45.5%)	100 (49.0%)	59 (52.7%)	84 (47.7%)	66 (35.3%)		
Current smoker		59 (8.5%)	5 (2.5%)	3 (2.6%)	17 (9.4%)	34 (17.4%)	34.69 (3)	< 0.001
Alcohol use	No alcohol use	265 (37.4%)	102 (48.3%)	38 (32.5%)	68 (37.4%)	57 (28.6%)	20.25 (6)	0.002
	Non-risky drinking	343 (48.4%)	87 (41.2%)	64 (54.7%)	85 (46.7%)	107 (53.8%)		
	Risky drinking	101 (14.2%)	22 (10.4%)	15 (12.8%)	29 (15.9%)	35 (17.6%)		
Recreational drug use in last 6 months		19 (2.7%)	2 (1.0)	2 (1.7)	5 (2.8)	10 (5.0)	7.18 (3)	0.07
Number of medical	0	453 (63.9%)	155 (73.5%)	73 (62.4%)	118 (64.8%)	107 (53.8%)	18.54 (9)	0.03
conditions	1	195 (27.5%)	44 (20.9%)	33 (28.2%)	49 (26.9%)	69 (34.7%)		
	2	53 (7.5%)	10 (4.7%)	9 (7.7%)	13 (7.1%)	21 (10.6%)		
	≥ 3	8 (1.1%)	2 (0.9%)	2 (1.7%)	2 (1.1%)	2 (1.0%)		
Ever diagnosed with depression		214 (30.7%)	22 (10.7%)	21 (18.3%)	52 (28.7%)	119 (61.0%)	131.29 (3)	< 0.001
Initiated ART		691 (97.9%)	204 (97.6%)	116 (99.2%)	177 (97.8%)	194 (97.5%)	1.13 (3)	0.77
Last CD4 count	> 500	435 (69.2%)	119 (65.7%)	74 (70.5%)	122 (72.2%)	120 (69.0%)	4.56 (6)	0.60
(cells/mm^3^)	200–500	151 (24.0%)	51 (28.2%)	22 (21.0%)	34 (20.1%)	44 (25.3%)		
	< 200	43 (6.8%)	11 (6.1%)	9 (8.6%)	13 (7.7%)	10 (5.7%)		
Last HIV viral load	Undetectable	594 (88.1%)	170 (85.4%)	97 (89.0%)	163 (92.1%)	164 (86.8%)	4.45 (3)	0.22
	Detectable	80 (11.9%)	29 (14.6%)	12 (11.0%)	14 (7.9%)	25 (13.2%)		
Ever used HRT		54 (11.8%)	4 (3.3%)	9 (11.5%)	17 (14.1%)	24 (17.5%)	13.42 (3)	0.004

IQR: interquartile range; HRT: Hormone replacement therapy

Number with missing values: menopausal status: 9; ethnicity: 12; born UK: 11; employment status: 24; relationship status: 33; education: 30; smoking: 17; Initiated ART: 14; last HIV VL: 35; last CD4: 80; ever used HRT: 251

**Fig. 1:**
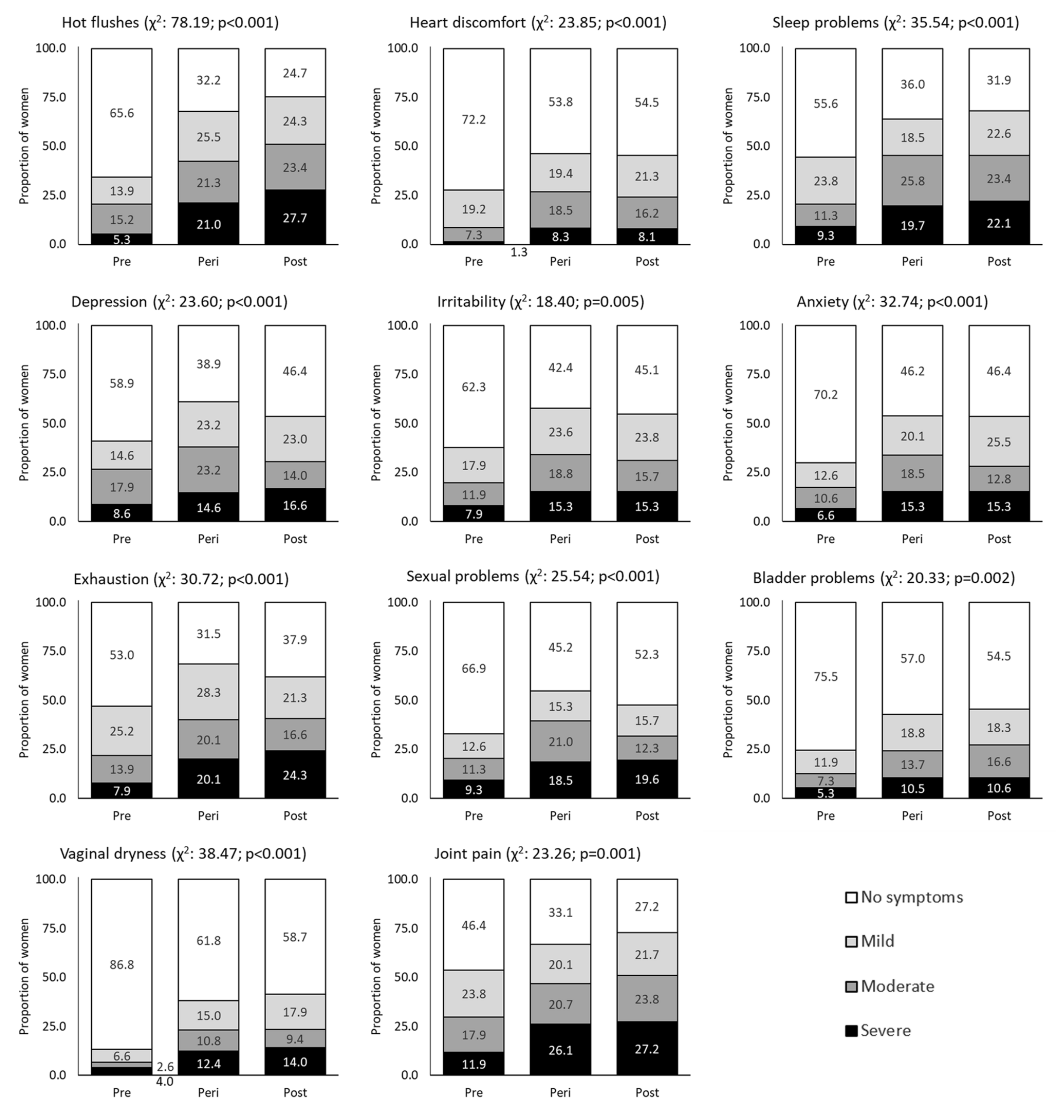
Severity of menopausal symptoms reported amongst women with a completed PRIME questionnaire and MRS rating scale score, stratified by menopausal status (n = 700)

Amongst those reporting menopausal symptoms, joint pain (66.4%) was most commonly reported, followed by hot flashes (63.0%), exhaustion (61.6%) and sleep problems (61.4%). Vaginal dryness (34.0%) was the least reported symptom. All symptoms were reported more commonly by peri- and post-menopausal women than those who were pre-menopausal (Fig. 1).

Overall, 211 (29.8%) women reported no/few symptoms, 117 (16.5%) reported mild symptoms, 182 (25.7%) moderate symptoms and the remaining 199 (28.1%) severe symptoms. Peri- and post-menopausal women were more likely to report more severe menopausal symptoms (as measured by the composite score) (Table 1). Symptom severity was associated with ethnicity, smoking status, number of medical conditions (in addition to HIV), alcohol use and depression.

For some symptoms (i.e. hot flashes, sleep problems, bladder problems, vaginal dryness and joint pain), the prevalence increased with menopausal stage, with peak prevalence in the post-menopausal group. However, for others (i.e. depression, irritability, exhaustion and sexual problems) the prevalence increased during peri-menopause but was lower in those who were post-menopausal (Fig. 1).

**Fig. 2:**
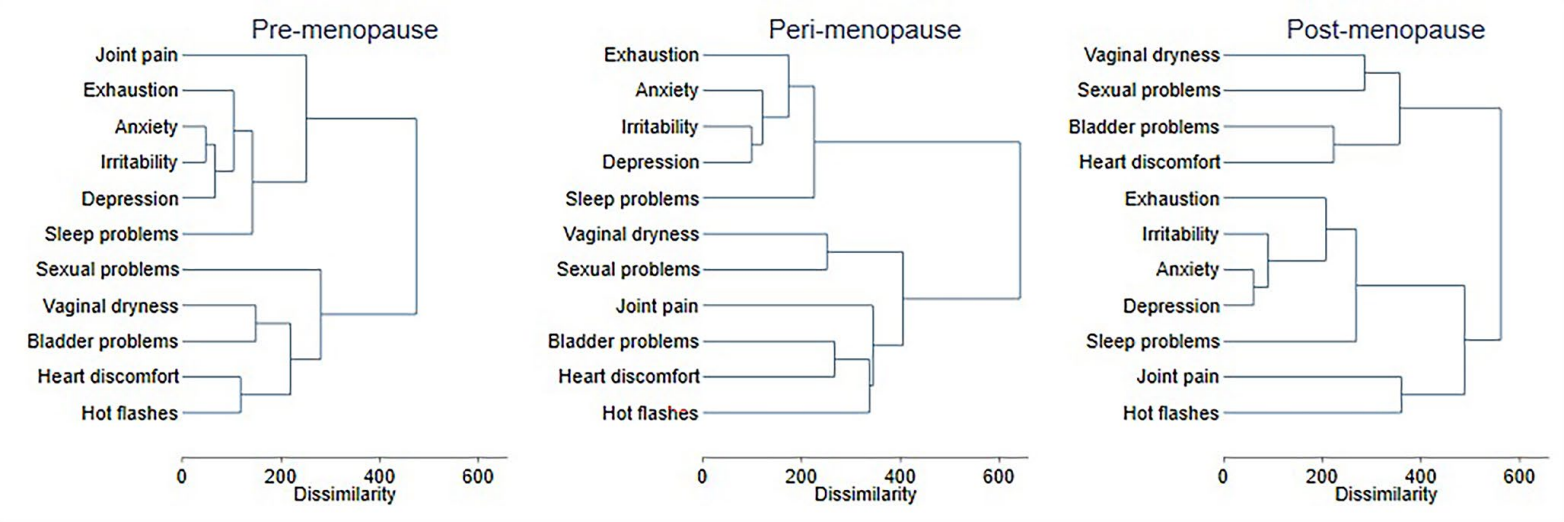
Dendrogram representing the clustering of menopausal symptoms reported by women in PRIME at each menopausal status *A shorter horizontal distance between clusters suggests a closer relationship between the symptoms

Our cluster analysis (Fig. 2) revealed that, amongst pre-menopausal women, there were two distinct clusters. The first included psychological symptoms (depression, irritability, anxiety and exhaustion) which clustered together with sleep problems and joint and muscular discomfort. The second comprised heart discomfort and hot flashes as well as vaginal dryness, bladder problems and sexual problems.

Women in the peri-menopausal stage reported a greater variety of symptoms. In this group, although psychological and sleep symptoms remained closely related, joint pain was now part of the second cluster. Within this second cluster, there were two clear subclusters: somatic symptoms (heart discomfort, joint pain and hot flashes) with bladder problems and sexual-related symptoms (sexual problems and vaginal dryness).

Among post-menopausal women, symptoms formed three distinct clusters. Psychological symptoms and sleep problems, urogenital symptoms and heart discomfort (with a clear relationship between vaginal dryness and sexual problems, and bladder problems and heart discomfort, respectively); and finally somatic symptoms (joint pain and hot flashes).

## Discussion

To the best of our knowledge this is one of the first studies to explore specifically which menopausal symptoms cluster together and change across pre-, peri- and post-menopausal status among women living with HIV. In initial analyses, we found approximately one-in-three women living with HIV aged 45–60 experienced *severe* menopausal symptoms, with severity peaking amongst those who were peri-menopausal. Our study suggests a high burden of potentially menopausal symptoms in women living with HIV aged 45–60 regardless of menopausal status, but with more pronounced symptoms among those who are peri- and post-menopausal. This high prevalence of menopausal symptoms has also been reported previously amongst women with HIV from studies based in the USA, Cambodia, Brazil, Nigeria and Thailand [13, 22–29].

The MRS has been adapted for use in a range of geographical settings [30]. However, there has been much debate about the variation in the reporting of severe menopausal symptoms (6.6% in Australia [31] vs. 34.9% in Spain [32]), potentially due to differences in cultural construction and understanding of menopausal symptoms [33, 34]. In the UK, the Medical Research Council (MRC) National Survey of Health and Development (a longitudinal study over the life course of women from England, Scotland, and Wales) reports the most recent data on the prevalence of menopausal symptoms in the general UK population [35]. We report a much higher prevalence of menopausal symptoms than that reported in the MRC study (e.g. hot flashes: 33.8% vs. 63.0%; joint pain: 48.1% vs. 66.4%; heart discomfort: 17.2% vs. 42.0%; sexual discomfort: 16.6% vs. 47.8%). While the MRC dataset provides the most representative symptom data amongst ageing women in the UK for comparison with women from the PRIME study, the study did not use the MRS to determine symptom severity.

In recent years, there has been greater emphasis on understanding how menopausal symptoms cluster in the general population. Ascertaining different patterns of menopausal symptoms may facilitate understanding of underlying the mechanisms [36, 37]. In general, studies have categorised women into three/four groups [38–43], which typically includes (i) women who experience a low level of or only few menopausal symptoms, (ii) women who experience moderate symptoms (somatic or psychological symptoms) and (iii) women with severe menopausal symptoms (particularly hot flashes). Only one study has attempted to disentangle the clustering of symptoms as women transition through menopause [42], with three clusters psychological, vasomotor/insomnia and pain) in the early menopausal stages, evolving into four clusters (psychological, pain/insomnia, vasomotor and breast pain/irritability) post-menopausally.

These studies focus on grouping women. To our knowledge, ours is the first study to explore the clustering of menopausal symptoms (rather than women) reported by women ageing with HIV using hierarchical agglomerative clustering techniques. Here, we report more distinct symptom clustering in later menopausal stages, particularly of urogenital and somatic symptoms. In contrast, psychological symptoms clustered similarly with sleep problems in all three menopausal stages considered.

Women living with HIV experience the additional challenge of distinguishing between symptoms of HIV, comorbidities, side effects of medication, and menopausal symptoms as they approach post-reproductive years. Although previous studies have highlighted how menopausal symptoms are under-recognized by women living with HIV and their healthcare providers [28, 44], the overlap of symptoms (including hot flashes/night sweats, exhaustion, sleep problems and depression) may give us an understanding as to why current evidence suggests over half of primary care providers are concerned about missing a HIV-related illness when managing menopause in this patient group [45]. By identifying the emergence of distinct clusters of urogenital (vaginal dryness, bladder problems and sexual problems) and somatic (hot flashes and joint pain) symptoms in peri- and post-menopausal women living with well-controlled HIV, our study provides confidence to women and healthcare providers in attributing the co-occurrence of these symptoms to reproductive ageing. Furthermore, these findings will help healthcare providers to elicit a comprehensive symptom history (by proactively assessing for symptoms that are known to co-occur) and facilitate appropriate assessment and management.

A notable finding is the persistent clustering of psychological symptoms with sleep problems through all menopausal stages amongst women with HIV. This highlights the enduring presence of mood and sleep-related symptoms in women living with HIV aged 45–60 *regardless* of menopausal status. The impact of a HIV diagnosis on psychological and/or sleep problems [46–51], and the further impact of these symptoms on HIV outcomes [52–54] have been well established. Therefore, our findings underline the importance of proactively assessing psychological and sleep symptoms and providing key mental health services for all midlife women living with HIV.

To date, PRIME is the only study in the United Kingdom providing a large, representative sample of women, and using validated tools, to understand the menopause transition amongst those living with HIV. However, it is important to acknowledge some key limitations of our study: by using hierarchical agglomerative clustering we were able to visualise the correlation between menopausal symptoms that co-occur. There is debate on how to define specific clusters and therefore a restriction of this methodology is the inability to quantify the proportion of women who fall into each of these clusters. Further research to describe the prevalence of these clusters would therefore be valuable. Unfortunately, there were limited or no data available on some factors which are known to be associated with the presence of menopausal symptoms (body mass index, polycystic ovary syndrome or treatment of menopausal symptoms i.e. non-hormonal hot flash treatments including selective serotonin reuptake inhibitors and gabapentanoids), and therefore could not be considered in this study. The MRS scale, although validated in the general population, has not yet been validated specifically in women living HIV. As previously discussed, the overlap between menopause- and HIV-related symptoms means that we cannot be certain that MRS is capturing menopausal rather than HIV-related symptoms. It is also important to note that we used self-reported menstrual pattern to determine menopausal status. Although this is a method widely used in the general population, there is a risk of misclassifying menopausal status. Additionally, women who reported to have ever been diagnosed with depression were less likely to complete the MRS questionnaire. Therefore, this study may be underestimating issues related to psychological symptoms. Finally, this cross-sectional analysis does not allow us to determine changes in symptoms in individuals over time, highlighting the important need for longitudinal data.

## Conclusions

In conclusion, drawing upon data from a large, representative sample of women living with HIV aged 45–60 in England, we have identified a high burden of menopausal symptoms across all domains. Although the MRS cannot differentiate between menopause and HIV-related symptoms, the increasing severity of symptoms in peri- and post-menopause as well as the emergence of distinct clusters of urogenital and somatic symptoms, point to the role of reproductive ageing.

Of note, psychological and sleep symptoms are prevalent and cluster together throughout all three menopausal stages. We therefore recommend regular and proactive assessment of potentially menopausal symptoms in women living with HIV aged 45–60 [55], with an awareness of how certain symptoms may co-exist (and specifically enquiring about them) and of the persistence of psychological and sleep symptoms regardless of menopausal status. Furthermore, providing women living with HIV with more nuanced information on menopausal symptoms, and how they may evolve over the transition, may enable them to be better prepared and empowered to seek appropriate support. In eliciting a clear and comprehensive account of symptoms, and through providing detailed information to women, healthcare providers will be better able to support women living with HIV to optimize quality of life as they age and advocate for better pathways to mental health services, psychology, and peer support.
